# Postmortem coronary artery calcium score in cases of myocardial infarction

**DOI:** 10.1007/s00414-021-02586-z

**Published:** 2021-04-13

**Authors:** Katarzyna Michaud, Virginie Magnin, Mohamed Faouzi, Tony Fracasso, Diego Aguiar, Fabrice Dedouit, Silke Grabherr

**Affiliations:** 1 University Center of Legal Medicine Lausanne-Geneva, Chemin de la Vulliette 4, CH - 1000 Lausanne 25, Switzerland; 2 Lausanne University Hospital, University of Lausanne, Lausanne, Switzerland; 3 Division of Biostatistics, Center for Primary Care and Public Health (Unisanté), Lausanne, Switzerland; 4 Geneva University Hospital, University of Geneva, Geneva, Switzerland; 5 Department of Forensic Pathology, Rangueil University Hospital, Toulouse, France

**Keywords:** Sudden cardiac death, Coronary calcifications, Coronary artery calcium score, Postmortem imaging

## Abstract

Sudden cardiac death (SCD) related to atherosclerotic coronary artery disease (ACAD) resulting in myocardial infarction is the most prevalent cause of death in western countries. In clinical practice, coronary artery calcium score (CACS) is considered an independent predictor of coronary events, closely related to atherosclerotic burden and is quantified radiologically by the Agatston score being calculated through computed tomography. Postmortem computed tomography (PMCT) allows the visualization and quantification of coronary calcifications before the autopsy. However, it was reported that some patients who died from severe ACAD had a zero CACS in PMCT. In this study, a retrospective evaluation of CACS in adult’s myocardial infarction cases related to ACAD, with available CACS and histological slides of coronary arteries, was performed in order to gain a deeper understanding of coronary calcifications and their role in myocardial infarction cases. The CACS was calculated by using the software Smartscore 4.0 after the radiological examination on a 64-row CT unit using a specific cardiac protocol. Thirty-six cases were identified out of 582 autopsies, recorded during a 2-year study period (29 men, 7 women; age 56.3 ± 11.7). CACS was 0–10 in 5 cases (5 men, 44.8 ± 13.7), 11–100 in 8 cases (6 men, 2 women, 53.1 ± 7.7), 101–400 in 13 cases (11 men, 2 women, 57.4 ± 9.6), and > 400 in 10 cases (9 men, 1 woman, 63.1 ± 11.9). Coronary thrombosis was found in 28 cases, histologically identified as plaque erosions in 6 cases and as plaque ruptures in 22 cases. Statistical analyses showed that CACS increases significantly with age (*p*-value < 0.05) and does not show significant correlation with gender, body weight, body mass index, and heart weight. CACS was significantly higher in plaque ruptures than in plaque erosions (*p*-value < 0.01). Zero or low CACS on unenhanced PMCT cannot exclude the presence of myocardial infarction related to ACAD. This paradoxical discrepancy between imaging and autopsy findings can be explained considering the histological aspect of fatal coronary plaques.

## Background

Sudden cardiac death (SCD) related to atherosclerotic coronary artery disease (ACAD) resulting in myocardial infarction is the most prevalent cause of death in western countries [1, 2]. ACAD can explain the death when autopsy reveals the presence of an acute coronary thrombosis and can be considered the cause of death when the circumstances and clinical history suggest an SCD and autopsy demonstrates severe coronary stenosis (>75%), with or without histological signs of myocardial ischemia and after exclusion of any other cause of death [3]. These autopsy findings correspond to the types 1 and 2 of updated clinical definition of myocardial infarction [4, 5]. The underlying pathology of mural or occlusive coronary thrombosis is variable, and can be due to plaque ruptures, erosions, or, less frequently, protruding calcified nodules [6–8].

Coronary calcifications are associated with ACAD and cardiac CT can provide quantification of coronary calcification. The radiological grading of ACAD is based on Agatston score, and classified as no evidence of ACAD (0 calcium score), minimal (1-10), mild (11-100), moderate (101-400), and severe (>400). Coronary artery calcium score (CACS) is considered in clinical practice as an independent predictor of ACAD events and has been found to be a marker of vascular lesion that correlates closely with overall atherosclerotic burden [9–12]. A zero CACS is considered the most powerful negative risk factor for development of a coronary event and the assessment of CACS appears to be the most predictive in the intermediate-risk group according to clinical scorings [13, 14].

Imaging techniques have become essential in postmortem investigations, especially in forensic practice. Among them, postmortem computed tomography (PMCT) is the most widely accessible and the most frequently used. PMCT allows the visualization and quantification of coronary calcifications before the autopsy. There are many controversies about how to interpret coronary calcifications on PMCT and how to interpret their presence for cases of sudden death [3, 15–17]. Some recent postmortem studies demonstrated that Agatston scoring zero or low cannot rule out the presence of extensive stenosis [16] and that CACS can neither confirm nor exclude death due to ACAD [18]. In a recent Australian study, it was reported that about one-third of patients who died from severe ACAD had a zero CACS in postmortem imaging [19]. This paradoxical discrepancy between imaging and autopsy findings has not been investigated nor explained. The goal of the study was firstly to assess the CACS in cases of autopsy-proven myocardial infarction cases. Secondary, the CACS was analyzed in different types of fatal coronary plaques in order to improve the understanding and interpretation of coronary calcifications detected by imaging and to explain why CACS zero or low cannot rule out the presence of extensive stenosis/or thrombosis.

## Material and methods

A retrospective evaluation of CACS was performed in cases of adults’ sudden death cases attributed to myocardial infarction related to ACAD diagnosed after a medico-legal autopsy. We selected cases in which PMCT with a special protocol permitting CACS evaluation was available as well as histological examination of coronary arteries was performed. The autopsies were conducted during a 2-year period (2017 and 2018) according to international guidelines [3, 20]. Selected were cases with an acute coronary thrombosis or with ACAD associated with stenosis > 75% and myocardial ischemia at histological or immunohistological examination, without any other significant pathological and toxicological findings, corresponding to the updated clinical definition of myocardial infarction types 1 and 2 [4, 5]. Cases showing putrefaction, carbonization, and traumatic injuries of the heart (not related to resuscitation attempts) and cases after percutaneous coronary revascularization procedures and/or coronary artery bypass grafting were excluded. Cases with concomitant pathological lesions, playing potentially a role in the death, or with a concomitant cause of death, such as acute intoxication were also excluded. After an external examination and a full body PMCT completed with a postmortem cardiac CT, a full autopsy was performed. Segments of coronary arteries, which appeared occluded or otherwise pathological during autopsy, were collected for histological examination. Comprehensive toxicological investigation was performed using urine immunoassays and gas chromatography-mass spectrometry (GC–MS) on peripheral blood and urine for cases without coronary thrombosis, to exclude another cause of death.

### Radiological examination

**Fig. 1:**
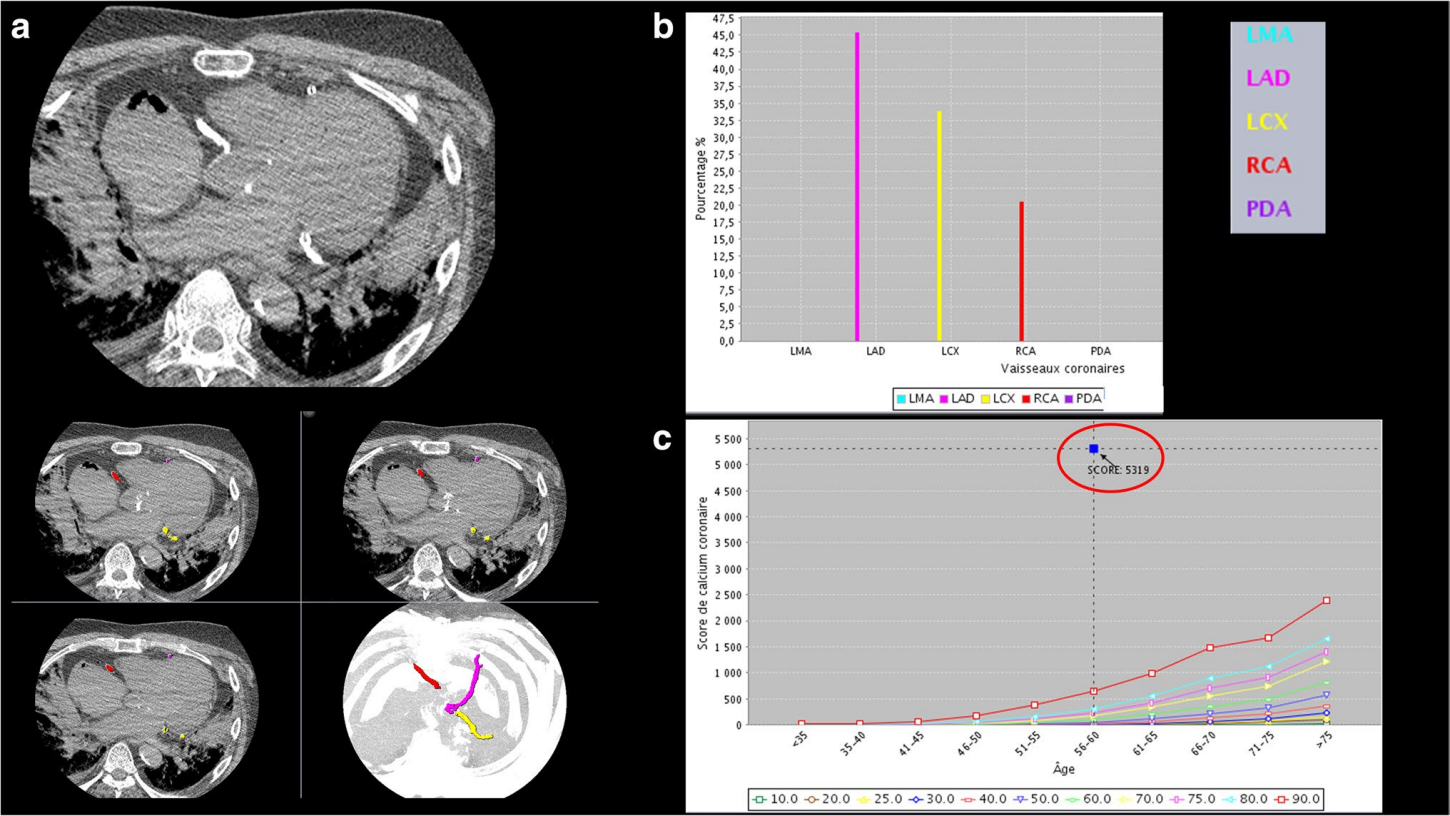
Measurement of CACS. Case of a 56-year-old man known for chronic ischemic disease. The total CACS was 5319. Image analysis was performed using the software Smartscore 4.0, running on the GE Advantage Windows Workstation. **a** When selecting a vessel name from the list, a separate calcium score is generated for each vessel, as well as a total calcium score. The color varies according to the vessel selected (in pink LAD, left ascending artery; in yellow LCX, left circumflex artery; in red RCA, right coronary artery). **b** Percentage of the calcifications for each scored vessel. **c** The graph shows the total CACS (blue square in the red circle) and places the patient into a percentile rank, based on the population database. Each colored line represents an age range. LMA-left marginal artery, LAD- left anterior descending, LCX- left circumflex artery, PDA- posterior descending artery

The radiological examination was performed on a 64-row CT unit (CT LightSpeed VTC; GE Healthcare, Milwaukee, USA). A full body helical scan was performed, and completed with a specific cardiac CT protocol with a non-enhanced sequential acquisition mode for each case. This scanning protocol had a display field of view of 25.0 cm, tube voltage of 120 kV, and X-ray tube current of 400 mA. The CACS was retrospectively calculated for each case by using the software Smartscore 4.0 of an Advantage Windows server (GE Healthcare, Milwaukee, USA) using a standard Agatston/Janowitz method. The CACS was assessed for each coronary artery and as total score by a board-certified radiologist trained in postmortem imaging (Fig. 1).

### Statistical method

Cases were classified into four groups according to their CACS: 0–10, 11–100, 101–400, and > 400. The data obtained for different groups of CACS were summarized in Table 1 by the mean (± SD, standard deviation) for continuous variables and by the number for categorical ones. Association between CACS groups and age, body weight, and heart weight was performed using the non-parametric Kruskal–Wallis test. For the gender and the presence of thrombosis, the Fisher’s exact test was used. Statistical analyses were performed using the STATA software by a senior statistician (one of the authors) [21].

**Table 1:** Summary and comparison of CACS groups by gender, age, body weight, heart weight, and thrombus (*CACS* coronary artery calcium score; *E* eroded plaques; *R* ruptured plaques)

CACS	Gender *n* (m/f)	Age (years) mean ± SD (min–max)	Body weight (kg) mean ± SD (min–max)	Heart weight (g) mean ± SD (min–max)	Thrombus *n* (E/R)
0–10	5 (5/0)	44.8 ± 13.7 (29–57)	82.2 ± 16.8 (64–103)	438.8 ± 56.4 (369–525)	3 (3/0)
11–100	8 (6/2)	53 ± 7.7 (43–67)	90.8 ± 13.2 (74–120)	449.4 ± 52.1 (380–505)	5 (1/4)
101–400	13 (9/4)	57.4 ± 9.6 (46–76)	86.5 ± 18.9 (59–130)	465.4 ± 202.2 (255–1025)	11 (2/9)
> 400	10 (9/1)	63.1 ± 11.9 (49–85)	88.6 ± 9.52 (69–106)	527 ± 137.2 (360–750)	9 (0/9)
All	36 (29/7)	56.3 ± 11.7 (29–85)	87.4 ± 14.97 (59–130)	475.3 ± 144.6 (255–1025)	28 (6/22)
*p*-value	0.47^*^	0.02^#^	0.77^#^	0.41^#^	0.005^*^

^*^Fisher-exact test

^#^Kruskal–Wallis test

## Results

A total of 582 autopsies were performed during the study period and 36 cases (29 men, 7 women; age 56.3 ± 11.7) fulfilled the inclusion criteria. CACS was 0-10 in 5 cases (5 men, 44.8 ± 13.7), 11-100 in 8 cases (6 men, 2 women, 53.1 ± 7.7), 101-400 in 13 cases (11 men, 2 women, 57.4 ± 9.6), and more than 400 in 10 cases (9 men, 1 woman, 63.1 ± 11.9). The age was higher for women (60.6 ± 9.1, min = 49 and max = 76) in comparison to men (55.2 ± 12.4, min 29 and max 85) but not statistically different (*p* = 0.21, two-sample Wilcoxon rank-sum (Mann-Whitney) test). Acute coronary thrombosis was found at autopsy in 28 cases. Coronary thromboses were then identified and classified histologically as an erosion in 6 cases and as a ruptured plaque in 22 cases. In 16 cases, the thrombosis was found in the right coronary artery (13 ruptures and 3 erosions), in 9 cases in the left anterior descending artery (6 ruptures and 3 erosions), and three ruptured plaques in the circumflex artery, all were situated in the proximal segments. The toxicological analyses, performed for cases without coronary thrombosis, were negative.

**Fig. 2:**
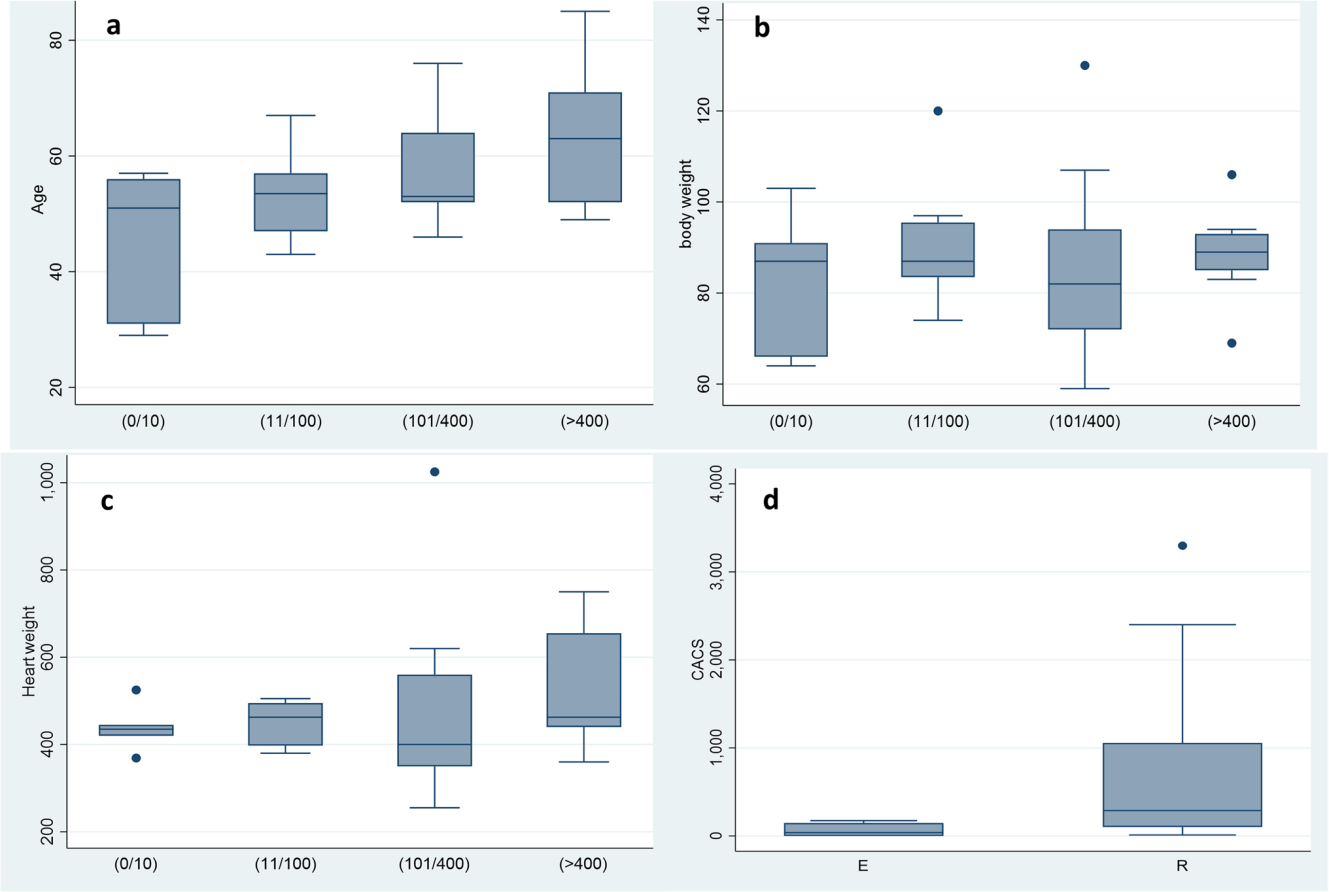
Distribution of CACS as a function of the age (**a**), body weight (**b**), heart weight (**c**), and in different type of coronary plaques (**d**); E: erosion; R: rupture

**Fig. 3:**
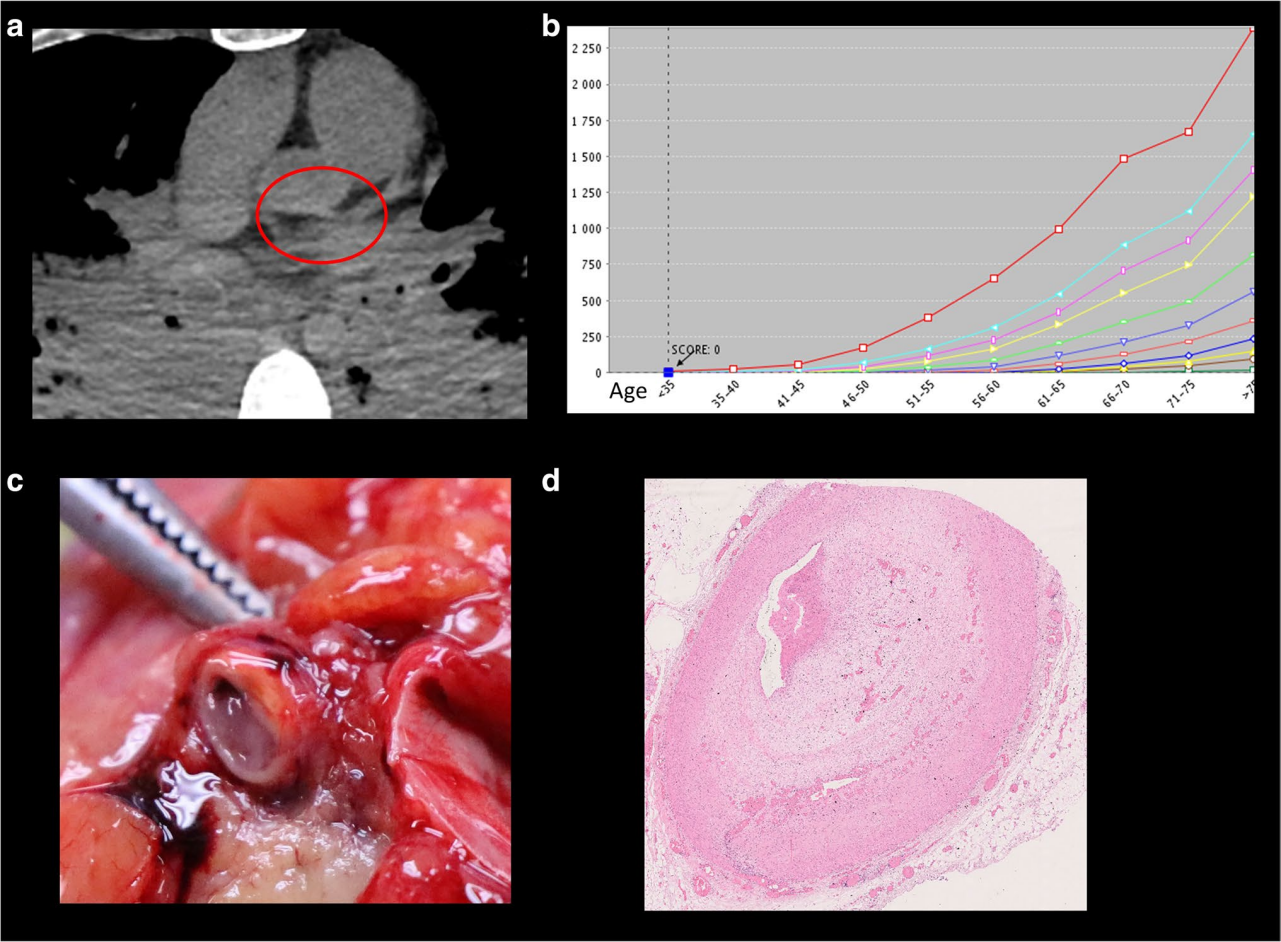
Coronary plaque erosion. A 30-year-old man complaining for some weeks of pain in the left arm and found dead at home. **a** PMCT without calcification on the proximal part of the anterior interventricular artery (red circle). **b** CACS for this case was zero. **c** Macroscopic view of a thrombosis on plaque of the proximal part of the left anterior descending artery. **d** Histologic slide showing a subocclusive thrombosis on an eroded plaque (H&E staining)

**Fig. 4:**
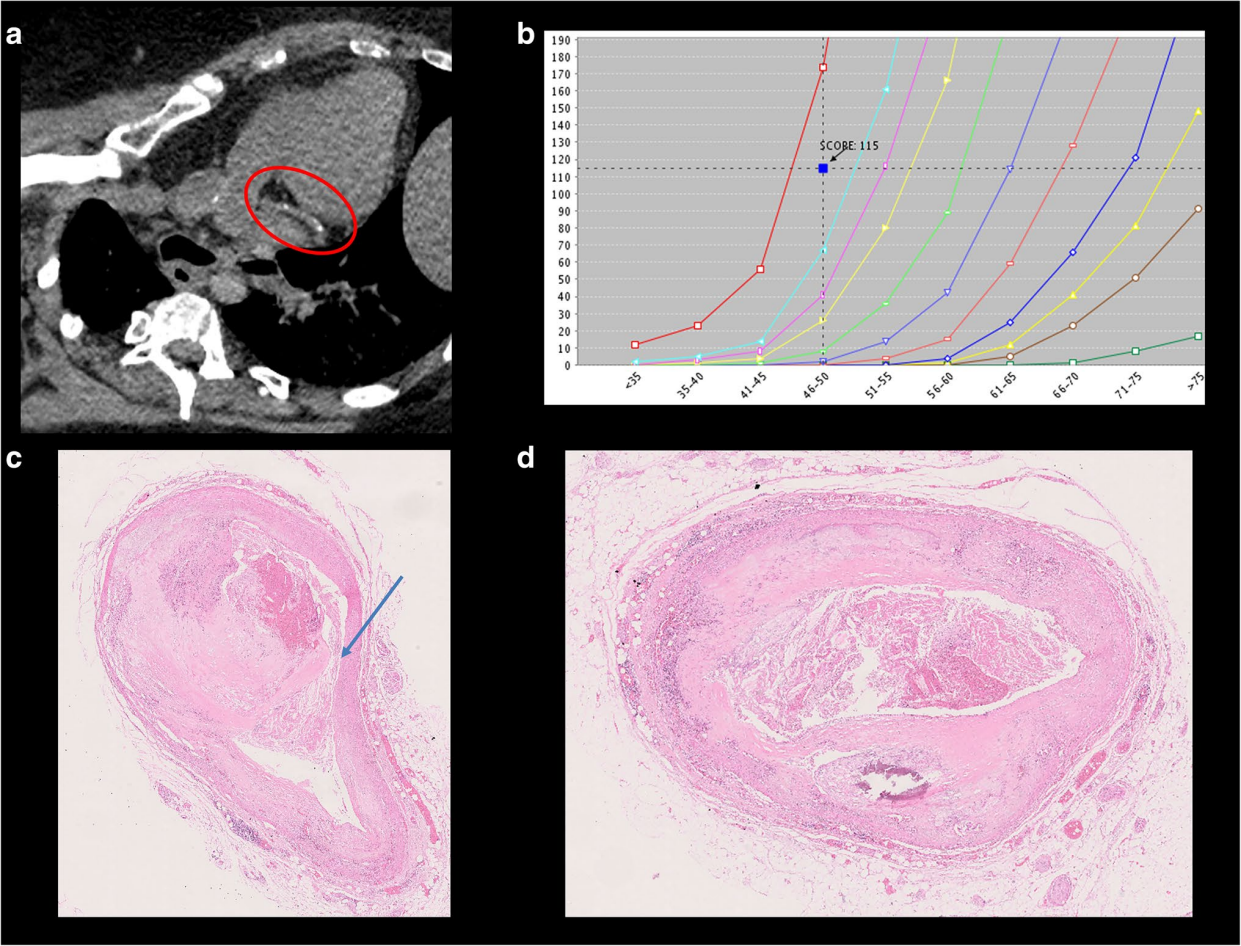
Coronary plaque rupture. A 49-year-old woman found dead home, known for hypertension and obesity. **a** PMCT with some calcifications on the circumflex artery (red circle). **b** CACS was 115, above the reference value. **c** Histology of the circumflex artery showing the rupture of the plaque (blue arrow) and protrusion of the thrombotic material into the lumen (H&E staining). **d** Thrombotic material in the lumen of the circumflex artery, some calcifications are observed in the wall (H&E staining)

The results of statistical analyses are presented in the Table 1. CACS increase significantly with the age (*p*-value < 0.05); there were no significant differences for the gender, body weight, body mass index, and heart weight. CACS was significantly higher in cases presenting ruptured thrombotic plaques than in erosions (*p*-value < 0.01). The mean CACS in cases with eroded and ruptured plaques were respectively 66.1 and 685.9. The results are presented in Table 1 and Figs. 2, 3, and 4.

## Discussion

Although vascular calcification is a well-known hallmark of atherosclerosis, the relationship between the extent of coronary calcifications, acute coronary syndrome, myocardial infarction, and SCD remains complex and partially unknown.

In our study, we observed that in about one-third of cases, postmortem CACS corresponded to atherosclerotic burden considered clinically as zero or minimal/mild. In another third of cases, CACS was moderate. This is finally not surprising if we consider the current knowledge concerning the pathophysiology of atherosclerosis and coronary thrombosis resulting in SCD, especially in young patients [8, 22–24]. Some histopathological studies showed that over a half of coronary atherothrombotic lesions leading to sudden death show rare or no calcification, whereas calcified nodules rarely lead to occlusion [24, 25]. However, up to now, no study was performed evaluating postmortem CACS in histologically different types of coronary plaques. The mural or occlusive coronary thrombosis resulting in SCD is due essentially to plaque ruptures, erosions, or less frequently calcified nodules. The majority of eroded plaques are described less calcified than ruptured ones, although microcalcifications are observed in approximately 40% of such lesions [24, 26]. The percentage of eroded plaques varies in the literature but can be observed in about 40–50% of young SCD who died from ACAD [27–29]. Therefore, our postmortem radiological and pathological results, showing that eroded plaques were significantly less calcified than ruptured plaques at postmortem imaging, are aligned with these previous histopathological studies.

On clinical CT images, coronary plaques are typically classified as calcified, partially calcified (mixed), and non-calcified plaques, according to the presence or absence of calcified components. It is known that about 4% of living patients, with an acute coronary syndrome, present non-calcified plaques with a zero CACS [24, 30, 31] (see also Fig. 3). For prediction of acute coronary syndromes, it is now considered more important to differentiate between plaques containing lipid-rich material and plaques with predominantly fibrous components [32]. Lesions leading to acute coronary syndrome and myocardial infarction often have a large necrotic lipid-rich core but the reliable differentiation between lipid-rich and fibrous lesion, made solely on the basis of CT attenuation, is not feasible [33]. Therefore, also in postmortem practice, the extension of coronary calcifications should be interpreted carefully. Even if coronary calcifications are easily detectable by PMCT, their detection is not sufficient to conclude that the death was consecutive to coronary occlusion and to myocardial infarction. A postmortem radiological study, performed in the UK, showed that CACS can neither diagnose nor exclude death due to ACAD [18]. A recent Australian study showed that presence of any coronary calcification in young population of patients under the age of 50 differed significantly between ischemic heart disease and non-ischemic deaths and that all cases with CACS > 100 had ischemic heart disease as the cause of death [19]. The authors observed however that 30.6% of patient who died from ischemic heart disease had a zero CACS but severe coronary artery disease at postmortem examination. Interestingly, these results are in accordance with our study. Unfortunately, the authors apparently did not perform histological evaluation for different plaques (erosions vs. ruptures) which could explain these results, as we evaluated in this study. In fact, in routine forensic practice, the thrombosed coronaries are not systematically collected and examined histologically.

It has been pointed out that, although Agatston CACS in clinical practice has improved the clinical ability to assess the clinical risk beyond traditional risk factors and risk scores, this score is imperfect [30, 34]. Some improvements were suggested, knowing that appropriate consideration of calcium density and its regional distribution could lead to an improved scoring system with a potential greatest predictive effect in patients presenting CACS score < 300. It was also emphasized that extensive local calcification is a marker of plaque stability and perhaps the use of statin therapy [34]. These clinical observations should be known by forensic pathologist and radiologists while interpreting PMCT before autopsy.

Finally, in our study, we observed that CACS increases with the age, which is in concordance with clinical studies showing steadily higher calcium amount and prevalence with increasing age [26, 29, 35]. We did not observe differences of CACS between men and women. This differs from studies on living patients and some histopathological studies [29, 35]. However, the number of female patients included in this study was low (7 cases/19.4%) and they were older than the male patients. Therefore, our findings could be compatible with clinical findings indicating that the progression of atherosclerosis and CACS in women occurs 10–15 years later in comparison with men [26]. This postmortem CACS correlation with the gender should be however verified in larger cohort studies. The number of cases included in this study was fairly small. Nevertheless, our preliminary data are so encouraging as to justify further postmortem investigations.

In conclusion, coronary calcifications represent one of the first postmortem information, visible in PMCT, for cases of presumed SCD. However, they should be interpreted carefully. Indeed, in the absence of coronary calcifications, SCD related to myocardial infarction can be misinterpreted on unenhanced PMCT, especially in cases with eroded plaques. Forensic pathologists and radiologists should evaluate very carefully all coronary arteries of sudden death victims, even if calcifications of coronary arteries are lacking or are not extensive in PMCT. In postmortem imaging of coronary arteries, visualization of the vessel’s lumen by injection of contrast medium is necessary [15, 28, 36–38]. More postmortem imaging and histopathological studies especially on distribution of coronary calcifications and on microcalcification are necessary to gain a deeper understanding of coronary calcifications and fatal coronary plaques in SCD cases.
